# Competitive strategy, development zone policy and firm growth: Empirical evidence from China

**DOI:** 10.1371/journal.pone.0292904

**Published:** 2023-10-18

**Authors:** Kaiyue Ye, Yugang Li, Peng Wu, Zhuhong Ye

**Affiliations:** School of Business, East China University of Science and Technology, Shanghai, China; Nanjing Audit University, CHINA

## Abstract

Competitive strategy plays an important role in achieving superior profits, but there is still much to be explored in terms of the effect on firm growth. This study focuses on exploring the relationship between competitive strategy and firm growth in emerging economies. We focus on how the development zone policies moderate this relationship. This study uses a two-way fixed-effect model to analyze data for 527 manufacturing firms listed in China’s Shanghai and Shenzhen A-shares from 2012-2021. Our empirical analysis showed that there is a significant positive relationship between low-cost strategy and firm growth and a significant negative relationship between differentiation strategy and firm growth. Compared with national development zones, firms in provincial development zones choose low-cost strategies that are more conducive to growth. Compared with provincial development zones, firms in national development zones choose differentiation strategies that are more conducive to growth. These findings contribute to understanding the mechanisms by which competitive strategy affects firm growth in different regional institutional contexts in emerging economies. The results of the study also have reference value for the government to optimize the development zone policies.

## 1. Introduction

The report of the 20th Party Congress states that it "insists on placing the focus of economic development on the real economy". The manufacturing industry is the main body of the real economy. The growth of manufacturing firms is not only a goal pursued by themselves but also an essential means for the State to achieve economic development. In an increasingly competitive global environment, Firms can grow by transforming their competitive strategies to improve their competitiveness [1]. The rapid growth of China Shengmu Organic Milk Limited from the start-up period to the steep decline in revenue after the IPO, and then to the gradual recovery in the past two years, is attributed to the sway of its low-cost strategy and differentiation strategy. Beijing-Hyundai Auto timely adjusted its strategy to make the product matrix continuously improved and finally gained market recognition. How to carry out a competitive strategy transformation and shift the drivers of business growth will be a key proposition to test the survival and development of every manufacturing firm [2, 3].

In the dominant competitive strategy paradigm, low-cost and differentiation have become the two basic strategies that firms can adopt to gain competitive advantage [4, 5]. The literature on the relationship between competitive strategy and firm growth has focused mainly on developed countries. Some scholars argue that low-cost strategies help firms gain cost leadership, occupy higher markets than competitors, and are more conducive to growth [6, 7]. Other scholars argue that firms implementing differentiation strategies compete with unique products and services, which can enhance sales revenue [8]. The studies that have been conducted on the relationship between competitive strategy and firm growth have not yet reached a consistent conclusion. Theoretical and empirical studies on the relationship between competitive strategy and growth, which focus on firms in emerging economies, are still weak.

Scholars have also been exploring the boundary conditions of the relationship between the impact of competitive strategy on firm performance to enrich the existing studies, such as social responsibility, innovation capability, and industry environment [2, 9, 10]. It was found that firms’ competitive strategy choice is conducive to improving firm performance when they match the external environment, and changes in the external environment also require firms to adjust their competitive strategies to remain adaptive [11]. Starting with Peng and Heath, firm growth in emerging economies has also received increasing attention in the last two decades [12]. Based on institutional theory, in countries with emerging economies, to adapt to the rapidly changing environment and respond to the challenges of institutional gaps, firms need to choose appropriate competitive strategies to gain an advantage for growth [13]. Firms often face system defects in the process of pursuing growth, such as imperfect intellectual property protection systems and lagging financial market development [14]. For emerging economies, the creation of a favorable institutional environment plays an important role in enabling firms to achieve growth by choosing appropriate competitive strategies [15].

As an institution with great Chinese institutional characteristics, development zones contribute to the construction and improvement of the local institutional environment and are an important vehicle for driving regional economic development [16]. The development zone is a specific area in which the country or region delimits a certain range for promoting the rapid development of the regional economy and implementing special policies and management means [17]. Its establishment is an important continuation of the special economic zone policy, which began with the establishment of the Dalian Economic and Technological Development Zone in 1984. Development zones are mainly divided into two levels: national development zone and provincial development zone [18]. National development zones are approved and managed by The State Council, and their establishment can often effectively reflect the regional development strategy at the national level. Provincial development zones are mainly approved by the municipal government, the provincial government, and the autonomous region government, and are more affected by the policy intention of the local government [19]. By 2021, there have been 634 national development zones and 2,094 provincial development zones.

Development zones are managed by different subjects, and thus the impact of different levels of development zones on firm behavior varies significantly [20]. Some studies have suggested that the agglomeration effect caused by the development zone policies can facilitate firms to acquire knowledge and technology spillover, thereby improving productivity and competitiveness, reducing production risks, and promoting faster growth of firms [17]. However, excessive competitive behavior among the development zones instead leads to a dampening effect on the growth of firms in the surrounding areas [21]. Current relevant studies have focused on the direct impact of development zones on firm growth [22], while the understanding of how firms’ competitive strategies are matched with different levels of development zones and how this matching affects firm growth remains limited. Given this, how do the different levels of development zones affect the relationship between competitive strategy and firm growth?

The remainder of the paper is shown below. The "Theoretical analysis and hypothesis development" section presents the theoretical foundations and hypothesis derivation. The "Data, variables and methodology" section describes the sample selection, variable descriptions, and model specification. The section "Empirical results" describes the empirical results. The "Conclusion and discussion" section summarizes the theoretical contributions, practical value, limitations and future research.

## 2. Theoretical analysis and hypothesis development

### 2.1. Competitive strategy and firm growth

Firm growth theory and resource-based theory are the most commonly used basic theories to explain firm growth. Firm growth theory assumes that multifunctional resources enable firms to restructure resources in novel ways to create growth [23]. The resource-based theory emphasizes that firms with a large number of valuable, rare, imperfectly imitable, and non-substitutable resources (VRIN resources) will be more likely to gain sustained competitive advantage and achieve growth [24]. Related scholars have focused more on the application of resource-based theory in explaining firm growth, instead ignoring the fact that the resource characteristics mentioned by firm growth theory are not the same as those emphasized by resource-based theory [5, 8]. The study points out that growth is a unique performance outcome of the firm and the resource-based theory based on VRIN resources does not explain it well. The relationship between multifunctional resources and firm growth is more closely aligned with firm growth theory [25].

Competitive strategy is one of the most important strategies for firms. Low-cost strategy refers to a firm’s strategy of creating a low-cost advantage over peers to achieve operational efficiency and increase market share. Differentiation strategy refers to a firm’s efforts to capture the market by offering unique products or services that focus on innovation, customer satisfaction, and brand image [26]. Porter’s competition theory suggests that firms should focus on low-cost strategies or differentiation strategies to produce superior performance. It proposes that five forces in the industry determine the size and degree of competition, which are the competitiveness of existing competitors in the same industry, the ability of potential competitors to enter, the substitution ability of substitutes, the bargaining power of suppliers, and the bargaining power of buyers [4].

#### (1) Low-cost strategy and firm growth

From a resource perspective, multifunctional resources facilitate the combination and wide application, providing firms with the means to develop new markets. Such resources have low transaction costs and also allow for rapid transfers, enabling firms to adapt to changing environments and quickly seek to identify new opportunities [12, 23]. To save costs, firms implementing low-cost strategies make the best use of their remaining resources and redirect resources from one purpose to new and more efficient activities. They enhance their resource mix capabilities by skillfully integrating resources and producing affordable substitutes to achieve growth [25, 27]. Given this, firms implementing low-cost strategies can grow by leveraging multifunctional resources and relying on cost advantages. In countries with emerging economies, firms are more inclined to identify and refine new value from existing resources to implement low-cost breakthrough innovations [28, 29]. Low-cost breakthrough innovation highlights low-cost strategies as the basis for achieving major innovations and breakthroughs in products and technologies. Firms focus on breakthrough innovation as the basis for integrating attributes such as low cost, design thinking, openness, and inclusiveness to provide consumers with better-quality products and services [30]. Given this, firms implementing low-cost strategies can rely on cost advantages to achieve growth by fully combining and piecing together multifunctional resources [31].

From a competitive perspective, the low-cost strategy requires firms to emphasize the vigorous pursuit of cost reduction along the value chain and to continuously increase production capacity below competitors’ costs [32]. First, firms that implement low-cost strategies rely on economies of scale and purchase large quantities of raw materials. Firms are more competitive in bargaining with upstream suppliers and can be motivated to leverage cost advantages for growth [33]. Second, emerging economies have a large number of low-income earners and more price-sensitive customers. Low-cost strategies can increase buyer retention through lower prices, which positively affects firm growth. Third, firms implementing a low-cost strategy have a cost advantage over their competitors, giving them a better chance of surviving and capturing more market share when they sell their products at lower prices than their competitors [34]. In summary, low-cost strategies can provide consumers with the opportunity to purchase products at low prices, which increases sales revenue and contributes to firm growth [35]. The Galanz Group implemented a low-cost strategy in the market for microwave ovens and other small home appliances. The scale of production of microwave ovens was much higher than that of its competitors, and the rapid expansion of production scale brought about a significant reduction in production costs. It has built up business barriers by price and dominated absolutely in the market.

Based on the above discussion, we propose the following hypothesis:

Hypothesis 1 (H1): The implementation of a low-cost strategy has a significant positive effect on firm growth.

#### (2) Differentiation strategy and firm growth

Based on the resource perspective, the differentiation strategy relies primarily on product or service uniqueness and customer loyalty to build competitive advantage [36]. Firms implementing differentiation strategies are committed to tapping VRIN resources and tend to engage in product innovation and exploratory innovation to provide better products or services [37]. However, most of the firms in China are mainly catch-up types, with backward technology levels, and face great difficulties in obtaining VRIN resources. Firms try to improve sales channels and product quality by imitating innovations [38]. The cost of such imitation may be higher than the cost of establishing these resources and capabilities by firms that already have a competitive advantage. When consumers are more price-sensitive, they may voluntarily give up the uniqueness of the product or service, to the detriment of the firm’s sales revenue. In addition, there is often an inherent tension between the pursuit of profit and growth, especially in countries with emerging economies, where the scarcity of resources makes firms prioritize the pursuit of profit to survive [14]. Firms that implement differentiation strategies are more likely to reap profit once they have access to VRIN resources and are different from other firms in terms of brand image and customer service [39]. Therefore, with limited resources, Chinese firms will maximize VRIN resources as they differentiate themselves, which can be profitable but difficult to achieve growth.

Based on the competitive perspective, manufacturing firms go through various stages from suppliers to buyers from the production, and processing to the sale of products [4]. Currently, the market is characterized by rapidly changing consumer demands and increasing substitutability of homogeneous substitutes. This makes the market very competitive. According to Porter, firms should define their relative position in the competition for investments to gain a lasting competitive advantage [40]. Most firms implementing differentiation strategies are more likely to encounter strong competitors as they overlap with foreign MNCs in terms of market positioning [41]. The implementation of a differentiation strategy requires a certain level of innovation capability. The higher the level of differentiation of a firm when the technological innovation capacity is weak, but without surpassing foreign competitors, it is difficult for product or service uniqueness to attract consumers, thereby expanding market share the more adverse impact [42]. Throughout the existing domestic firms, a considerable part of the local firms can only survive with difficulty in the squeeze of various large foreign brands. Most brands are still dominated by foreign products. For example, in the cola market, almost Pepsi and Coca-Cola occupied the entire share. Cosmetics market, L’Oreal, Procter & Gamble, Unilever, and other foreign brands have also captured most of the market share. In practice, many firms in China are pursuing to enhance the level of differentiation. Some firms have been increasing R&D efforts and squeezing into the high-end market, threatening the position of foreign competitors and facing heavy obstacles to development. While some other firms also focus on the low- and mid-range markets, it is easier to occupy market share and achieve growth.

Based on the above discussion, we propose the following hypothesis:

Hypothesis 2 (H2). The implementation of a differentiation strategy has a significant negative effect on firm growth.

### 2.2. The moderating effect of development zone policies

As a typical example of institutional innovation in China, development zones have provided sufficient resources and a good institutional environment for firms in the zones since their establishment [43]. In policy practice, national development zones and provincial development zones are established with different fundamental objectives. The State Council manages national development zones, the establishment of which often effectively reflects the will for development at the national level. National development zones provide a good platform for innovation and require firms to take the lead in regional economic and local development. The construction of provincial development zones is the responsibility of local governments and is more influenced by local policies. The intensity of relevant preferential policies, such as land policies and tax subsidies, is not comparable to that of national development zones [44].

#### (1) The moderating effect of development zone policies on the relationship between low-cost strategy and firm growth

Firms that implement a low-cost strategy have the advantage of scale and can make profits in a relatively short time. These firms can employ more labor, solve social problems, and contribute to regional economic growth. They help local officials achieve their performance appraisal goals and are more likely to be supported by local governments. However, the low-cost strategy sacrifices the quality of the product or service to a certain extent and the competitive advantage it creates can be easily imitated. This traditional low-cost, high-energy-consumption growth model has drawn criticism from academics and is contrary to the purpose for which national development zones were established [37, 45]. On the one hand, firms in provincial development zones are more likely to be supported by local governments with low-cost resources such as land and capital. Firms that implement low-cost strategies have more energy to focus on improving portfolio capabilities, bringing into play the multifunctional characteristics of resources, and continuously improving the efficiency of resource utilization [46]. On the other hand, firms in provincial development zones tend to pursue scale advantages to be valued by local governments. Firms that implement low-cost strategies can reduce upstream and downstream channels and operating costs through the industrial aggregation effect. Firms are more cost-competitive, thus attracting consumers with lower prices and increasing their market share [17]. Therefore, compared with national development zones, firms in provincial development zones with low-cost strategies are more likely to receive support from local governments with low-cost resources. At the same time, to avoid innovation risks, firms will devote more energy to exploiting multifunctional resources and establishing cost advantages, which will have a positive effect on growth.

Based on the above discussion, we propose the following hypothesis:

Hypothesis 3 (H3). Compared with national development zones, firms in provincial development zones implement low-cost strategies that are more conducive to growth.

#### (2) The moderating effect of development zone policies on the relationship between differentiation strategy and firm growth

The implementation of a differentiation strategy by firms requires a lot of material and financial resources for R&D and innovation. This is highly likely to leave the firm with no revenue return in the short term. Inconsistency with consumer demand forecasts may also cause firms to experience failure in the implementation of the strategy [47]. However, firms implementing differentiation strategies are more sustainable in gaining a competitive advantage by continuously improving the quality of their products/services through innovation. It matches with the national development strategy and the state will provide quality resources for firm development [48, 49]. Therefore, firms in national development zones are more likely to be supported by quality resources such as national government subsidies, tax incentives, and talent. This is crucial for stimulating innovation and avoiding business risks [50, 51]. Firms that implement differentiation strategies can use sufficient information and quality resources to enhance the value of their products/services. They can respond to market changes and better meet consumer demand, which helps to promote growth [8]. In addition, the establishment of national development zones creates a favorable institutional environment for firm development and intensifies market competition among firms [20]. The crude growth model will accelerate the decline of firms. Firms that implement a differentiation strategy enhance core competitiveness through innovation, establish a good brand image, cultivate consumer loyalty, and gain a competitive advantage that is not easily imitated by competitors. Thus, they are more likely to survive in a competitive environment and occupy a certain market share [10].

Based on the above discussion, we propose the following hypothesis:

Hypothesis 4 (H4). Compared to provincial development zones, firms in national development zones implement differentiated strategies that are more conducive to growth.

## 3. Data, variables and methodology

### 3.1. Sample selection

We take Shanghai and Shenzhen A-share-listed manufacturing firms as the research objects. To obtain reliable data, the study subjects were screened by the following steps. (1) Select listed firms that have been in continuous operation during 2012-2021. (2) Excluding firms with abnormal operations (ST or PT). (3) Excluding firms with a gearing ratio greater than 100%. (4) Excluding firms with important data missing. (5) Excluding firms that belong to unclear development zones or are stationed in multiple development zones. Finally, we obtain a balanced panel dataset with a sample cross-sectional number of firms of 527 and observations of 5270. To eliminate the effect of outliers, the upper and lower 1% Winsorize shrinkage is done for all continuous variables. The data used are mainly from the WIND database, CSMAR data, and the China Development Zone Audit Bulletin Catalogue (2018 edition).

### 3.2. Variable descriptions

#### (1) Firm growth

Prior research has widely accepted sales revenue growth as the primary indicator of firm growth. Drawing on authoritative scholars [14, 52], we measure the annual percentage growth in sales revenue. A larger value indicates a faster rate of firm growth. The calculation formula is as follows.


Growth=(Salest‐Salest‐1)/Salest‐1×100%

#### (2) Competitive strategy

Based on domestic and foreign scholars’ research [53], combined with the financial indicators of domestic listed firms, we adopt the following way to measure competitive strategy. We use total asset turnover to measure the capital saving dimension of low-cost strategy and the ratio of sales revenue to cost of sales to measure the cost efficiency dimension of low-cost strategy [54]. By doing a principal component factor analysis on these two indicators, it was found that both indicators were attributed to a single factor and had similar eigenvectors (both factor loadings were 0.72). Therefore, we took the average of the two indicators as a measure of low-cost strategy. We used gross profit margin and operating expense income ratio to measure differentiation strategy [55, 56]. Similarly, after principal component analysis, it is found that both indicators are also attributed to a single factor with similar eigenvectors (both factor loadings are 0.92). We similarly took the average of the two indicators as a measure of differentiation strategy. Higher values indicate a higher degree of low-cost strategy or differentiation strategy of the firm.

#### (3) Development zone policy

National development zones mainly include economic and technological development zones, high-tech industrial development zones, export processing zones, bonded zones, Taiwanese investment zones, border cooperative economic zones, national tourist resort zones, and other types. There are two main types of provincial development zones. One type is economic development zones, with functions similar to national economic and technological development zones. One type is industrial parks, which focus on the development of various types of industrial projects. Referring to the studies of Alder et al. (2016) and Schminke and Van Biesebroeck (2013), we exclude development zones such as bonded zones, Taiwanese investment zones, and border cooperative economic zones, which are small in volume or whose industrial orientation is not manufacturing [16, 57]. We select the three most important types of national development zones: economic and technological development zones, high-tech industrial development zones, and export processing zones. Provincial development zones are dominated by development zones and industrial parks. These development zones are also important agglomerations of manufacturing firms [58].

Drawing on existing methods, the level of the development zone in which the firm is located is collected manually based on the registered address [50, 51]. The specific steps are as follows. ① For firms whose registered address information has development zones and industrial parks, directly find the name of the development zone from the "China Development Zone Audit Bulletin Directory (2018 Edition)" and identify the development zone level. ② For samples with difficult–to-identify address information, further compare the zip code information of the development zones and firms. At the same time, the information on the four ranges of national development zones is used as a supplement to identify the name and level of the development zone where the firm is located. ③ For firms that have not yet identified the name and level of the development zone, information is collected through the firm’s official website or the official websites of all development zones where the firm is located for identification. Through the effective combination of the above steps, the name and level of the development zone in which the firm is located can be accurately identified. A dummy variable is set to measure whether the firm is in a national development zone or a provincial development zone. The dummy variable is 1 if the firm is in a national development zone in the current year, and 0 if the opposite is true. Similarly, the dummy variable is 1 if the firm is in a provincial development zone in the current year, and 0 if the opposite is true.

#### (4) Control variables

Firm growth can be affected by a variety of factors. We select the firm size, firm age, financial leverage, profitability, sales margin, R&D intensity, shareholding ratio, independent director ratio, and institutional environment as control variables.

The full variable names and measurements are listed in Table 1.

**Table 1:** Variable names and measurements.

Variables	Name	Symbol	Measurement
Dependent variable	Firm Growth	Growth	Sales revenue year-on-year growth rate
Independent variables	Lowcost Strategy	Lowcost	Total assets turnover ratio The ratio of sales revenue to cost of goods sold
	Differentiation Strategy	Differ	Operating income expense ratio Gross Profit Rate
Moderating variables	National Development Zone	NDZ	1 if the firm is located in a national development zone, and 0 if not
	Provincial Development Zone	PDZ	1 if the firm is located in a provincial development zone, or 0 if not
Control variables	Firm Size	Size	Natural logarithm of total assets
	Firm Age	Age	Number of years on the market
	Financial Leverage	Lev	Gearing ratio
	Profitability	Roa	Return on Assets
	Sales Margin	Ros	The ratio of total profit to sales revenue
	R&D Investment	Rdi	The ratio of R&D investment to sales revenue
	Shareholding Ratio	Sr	The Shareholding ratio of the largest shareholder
	Independent Director Ratio	Dire	The ratio of independent directors to the total number of board of directors
	Institutional Environment	Index	Marketability Index

### 3.3. Model specification

Before processing the panel data, the Hausman test was first used to determine whether a random effects model or a fixed effects model should be used. As can be seen from the output of Table 2, the p-value corresponding to the Hausman test in both Model 1 and Model 2 is 0. The test results strongly reject the original hypothesis that the random effects model is the correct model. The fixed-effects model is applied in this paper.

**Table 2:** Hausman test results.

Variables	Model 1				Model 2			
	Fe	Re	Difference	S.E.	Fe	Re	Difference	S.E.
Lowcost	0.593	0.132	0.461	0.030				
Differ					-0.110	-0.025	-0.085	0.088
Size	6.928	0.169	6.759	0.884	4.162	-0.019	4.181	0.882
Age	-0.870	-0.611	-0.259	0.227	-0.936	-0.591	-0.345	0.231
Lev	0.196	0.157	0.039	0.031	0.276	0.176	0.010	0.031
Roa	1.051	1.020	0.031	0.058	1.591	1.145	0.446	0.053
Ros	0.004	0.005	-.001	0.000	0.004	0.005	-0.001	0.000
Rdi	1.401	0.886	0.515	0.198	0.978	0.615	0.363	0.203
Sr	-0.117	-0.115	-0.002	0.052	-0.077	-0.084	0.008	0.053
Dire	0.001	-0.043	0.044	0.066	0.011	-0.031	0.042	0.067
Index	-2.272	-0.782	-1.491	0.781	-2.055	-0.690	-1.364	0.790
Chi2(10)	373.05				139.97			
Prob>chi2	0.000				0.000			

## 4. Empirical results

### 4.1. Descriptive statistics

Table 3 presents the results of descriptive statistics for the variables, from which it can be seen that: (1) The maximum value of firm growth is 99.56, and the minimum value is -42.06, which indicates that there is a large gap in the growth of Chinese firms. (2) The maximum value of the low-cost strategy degree is 127, the minimum value is 8.634, and the maximum value of the differentiation strategy degree is 61.65, the minimum value is 0.906, which illustrates that there are significant differences in competitive strategies among firms. In addition, although there are more provincial development zones than national development zones, the number of firms in national development zones is greater than the number of firms in provincial development zones in the sample. This suggests that national development zones may be more conducive to sustainable firm development, which tends to be consistent with numerous scholars’ studies and further indicates that the sample data were chosen to be robust [20].

**Table 3:** Descriptive statistics.

Variables	N	Mean	Std. Dev.	Minimum	Maximum
Firm Growth	5270	11.080	23.010	-42.060	99.560
Lowcost Strategy	5270	39.200	21.810	8.634	127.000
Differentiation Strategy	5270	16.100	12.270	0.906	61.650
National Development Zone	5270	0.438	0.496	0.000	1.000
Provincial Development Zone	5270	0.193	0.395	0.000	1.000
Firm Size	5270	13.240	1.240	10.880	16.630
Firm Age	5270	14.400	5.154	3.000	27.000
Financial Leverage	5270	47.060	18.650	7.727	88.830
Profitability	5270	5.903	6.461	-13.930	27.830
Sales Margin	5270	-60.730	484.400	-3,535	839.500
R&D Investment	5270	2.547	2.294	0.000	10.690
Shareholding Ratio	5270	33.670	14.040	8.110	74.300
Independent Director Ratio	5270	36.850	5.232	30.000	57.140
Institutional Environment	5270	7.616	1.948	-1.420	11.400

Table 4 presents the Pearson correlation coefficients of the variables. It shows that low-cost strategy and differentiation strategy have a direct impact on firm growth. In addition, the variance inflation factors (VIF) are all less than 5, which indicates that there is no significant problem of multicollinearity among the variables. The above results tentatively indicate the existence of different patterns of influence relationships among the variables, and more accurate conclusions are subject to further empirical testing in later sections.

**Table 4:** Pearson correlations among main variables.

	(1)	(2)	(3)	(4)	(5)	(6)	(7)	(8)	(9)	(10)	(11)	(12)	(13)	(14)	VIF
(1) Growth	1														
(2) Lowcost	0.161***	1													1.37
(3) Differ	0.085***	-0.247***	1												1.56
(4) Ndz	-0.009	0.045***	-0.033**	1											1.30
(5) Pdz	0.035**	0.040***	-0.020	-0.431***	1										1.26
(6) Size	0.034**	0.069***	-0.104***	-0.008	0.012	1									1.47
(7) Age	-0.155***	0.003	0.028**	0.054***	-0.127***	0.290***	1								1.20
(8) Lev	0.021	0.114***	-0.394***	-0.005	0.024*	0.382***	0.040***	1							1.53
(9) Roa	0.314***	0.232***	0.414***	-0.038***	0.048***	0.098***	-0.064***	-0.302***	1						1.87
(10) Ros	0.222***	0.073***	0.124***	0.006	-0.010	0.004	-0.009	-0.120***	0.404***	1					1.21
(11) Rdi	0.029**	-0.268***	0.160***	0.113***	-0.058***	-0.039***	-0.046***	-0.162***	-0.019	0.0150	1				1.25
(12) Sr	-0.008	0.211***	-0.080***	-0.084***	0.048***	0.221***	-0.020	0.119***	0.089***	0.003	-0.135***	1			1.12
(13) Dire	-0.021	0.005	0.011	0.045***	-0.069***	0.077***	0.034**	0.002	-0.031**	-0.019	0.027*	0.033**	1		1.02
(14) Index	-0.060***	-0.027*	0.049***	-0.150***	0.080***	-0.011	0.124***	-0.122***	0.0220	0.026*	0.281***	-0.064***	-0.040***	1	1.18

## 4.2. Regression analysis

Table 5 presents the results of the regression analysis of the relationship between competitive strategy and firm growth. As shown in Model 1, among the control variables, firm size, financial leverage, profitability, sales margin, and R&D investment have a significant positive effect on firm growth. Firm age has a significant negative effect on the growth of the firm. Model 2 incorporates the low-cost strategy and model 3 incorporates the differentiation strategy. The results showed that the low-cost strategy had a significant positive effect on firm growth (β=0.575, p<0.001). The higher the degree of a firm’s low-cost strategy, the more beneficial it is to firm growth. Differentiation strategy has a significant negative effect on firm growth (β=-0.199, p<0.05). That is, the higher the degree of firm differentiation strategy, the slower the firm growth instead. The overall effect of the regression model is more satisfactory, and the above results verify hypotheses 1 and 2.

**Table 5:** The relationship between competitive strategy and firm growth.

Variables	Model 1	Model 2	Model 3
Lowcost		0.575*** (18.55)	
Differ			-0.199** (-2.32)
Size	4.450*** (5.13)	7.036*** (8.28)	4.473*** (5.16)
Age	-3.229*** (-13.03)	-3.156*** (-13.19)	-3.182*** (-12.80)
Lev	0.279*** (8.20)	0.203*** (6.15)	0.271*** (7.92)
Roa	1.373*** (19.12)	0.891*** (12.03)	1.416*** (19.11)
Ros	0.003*** (4.28)	0.004*** (5.47)	0.003*** (4.38)
Rdi	1.409*** (5.94)	1.761*** (7.66)	1.435*** (6.04)
Sr	-0.071 (-1.31)	-0.103** (-1.98)	-0.065 (-1.20)
Dire	0.019 (0.23)	0.012 (0.15)	0.019 (0.23)
Index	0.969 (1.26)	0.585 (0.78)	0.947 (1.23)
Year fixed effects	Yes	Yes	Yes
Adjusted R^2^	0.260	0.310	0.261

Note: ***, **, and * denote significance at the 1%, 5%, and 10% levels, respectively (two-tailed test). The p-values are in parentheses.

Table 6 presents the results of the moderating effect of the development zone level on the relationship between competitive strategy and firm growth. The results of the moderating effect of the development zone level on the relationship between low-cost strategy and firm growth are presented in models 1 to 3. In model 2, there is a significant positive relationship between low-cost strategy and firm growth (β=0.574, p<0.001), and the coefficient of the interaction term between national development zones and low-cost strategy is significantly negative (β=-0.103, p<0.05), which indicates the negative moderating effect of national development zones in the relationship between low-cost strategy and firm growth. There is a significant positive relationship between low-cost strategy and firm growth in model 3 (β=0.574, p<0.001), and the coefficient of the interaction term between provincial development zones and low-cost strategy is significantly positive (β=0.116, p<0.05), which indicates that there is a positive moderating effect between low-cost strategy and firm growth in provincial development zones. Hypothesis 3 was tested empirically. Compared to national development zones, firms in provincial development zones implement low-cost strategies that are more conducive to growth.

**Table 6:** The moderating effects of development zone policies.

Variables	Model 1	Model 2	Model 3	Model 4	Model 5	Model 6
Lowcost	0.575*** (18.55)	0.574*** (18.51)	0.574*** (18.51)			
Differ				-0.199** (-2.32)	-0.213** (-2.46)	-0.205** (-2.38)
Ndz		0.290 (0.14)			-0.690 (-0.32)	
Pdz			0.371 (0.18)			1.760 (0.81)
Ndz×Lowcost		-0.103** (-2.16)				
Pdz×Lowcost			0.116** (1.98)			
Ndz×Differ					0.219* (1.72)	
Pdz×Differ						-0.151 (-1.07)
Control variables fixed effects	Yes	Yes	Yes	Yes	Yes	Yes
Year fixed effects	Yes	Yes	Yes	Yes	Yes	Yes
Adjusted R^2^	0.310	0.311	0.311	0.261	0.262	0.261

Note: ***, **, and * denote significance at the 1%, 5%, and 10% levels, respectively (two-tailed test).

The p-values are in parentheses.

Models 4 to 6 present the results of the moderating effect of development zone level on the relationship between differentiation strategy and firm growth. In model 5, there is a significant negative relationship between low-cost strategy and firm growth (β=-0.213, p<0.05), and the coefficient of the interaction term between national development zones and low-cost strategy is significantly positive (β=0.219, p<0.01), which indicates that there is a positive moderating effect of national development zones in the relationship between differentiation strategy and firm growth. There is a significant negative relationship between differentiation strategy and firm growth in model 6 (β=-0.205, p<0.05), and the coefficient of the interaction term between provincial development zones and differentiation strategy is negative but not significant (β=-0.151, p>0.1), which indicates that there is no moderating effect between low-cost strategy and firm growth in provincial development zones. In summary, hypothesis 4 was tested empirically. Compared to provincial development zones, firms in national development zones implement differentiated strategies that are more beneficial to growth.

### 4.3. Robustness test

To ensure the reliability of the basic estimation results, this paper conducts a series of robustness tests. As presented in the robust regression results in Table 7, the new regression results are not substantially different from the previous paper.

**Table 7:** Robustness regression results.

Variables	Model 1	Model 2	Model 3	Model 4	Model 5	Model 6	Model 7	Model 8
Lowcost	0.546*** (16.94)	0.548*** (17.02)	0.549*** (17.00)				0.426*** (13.68)	
Differ				-0.205** (-2.29)	-0.220** (-2.44)	-0.211** (-2.34)		-0.178*** (-3.10)
Ndz×Lowcost		-0.123** (-2.53)						
Pdz×Lowcost			0.110* (1.77)					
Ndz×Differ					-0.235* (1.78)			
Pdz×Differ						-0.141 (-0.99)		
Control variables fixed effects	Yes	Yes	Yes	Yes	Yes	Yes	Yes	Yes
Year fixed effects	Yes	Yes	Yes	Yes	Yes	Yes	Yes	Yes
Adjusted R^2^	0.312	0.313	0.334	0.267	0.268	0.267	0.248	0.216

Note: ***, **, and * denote significance at the 1%, 5%, and 10% levels, respectively (two-tailed test).

The p-values are in parentheses.

#### (1) Changing the scope of the sample

The relevant literature suggests that when sampling is not uniform across regions, it may bias the estimation results. In this paper, we change the sample size and re-run the regression after deleting provinces with too many and too few sample firms.

#### (2) Adding omitted variables

The previous regression controls for year-fixed effects and firm-fixed effects, and although most firms do not change provinces and industries, this possibility is objective. To avoid this problem, we retain the year-fixed effects and individual-fixed effects, and further, add province-fixed effects and industry-fixed effects to re-run the regression.

#### (3) Changing the measurement method

The accuracy of the measure of firm growth is a key factor affecting the conclusion. In the robustness test, the regression is re-run by taking the log difference of sales revenue to measure the firm growth.

#### (4) Using instrumental variables to address the endogeneity issue

Due to path dependence, past firm strategies influence present strategies. Thus, the level of low-cost and differentiation strategies in past periods affects the degree of low-cost and differentiation strategies in the present, while firm growth in the current year is often directly influenced by the recent or current competitive strategies. To address possible endogeneity issues, this study performs two-stage least squares estimation (2SLS) with a three-period lag of a firm’s competitive strategy as an instrumental variable. The instrumental variables passed the indistinguishable test and the weak instrumental variable test, and the selection was reasonable. The predictor variables of endogeneity variables were obtained by regressing the independent variables using control variables and instrumental variables. The control and predictor variables were then selected as independent variables to perform a quadratic regression on the dependent variable.

## 5. Conclusion and discussion

### 5.1. Theoretical contributions

The possible theoretical contributions of this study are mainly reflected in the following two aspects. First, this study examines the relationship between competitive strategy and firm growth. We conclude that the low-cost strategy had a significant positive effect on firm growth. Differentiation strategy has a significant negative effect on firm growth. The studies on the relationship between competitive strategy and firm growth have focused mainly on developed countries. Theoretical and empirical studies targeting firms in emerging economies are still weak. We analyze panel data of Chinese listed manufacturing firms, expanding the empirical research on competitive strategy and firm growth. In the theoretical analysis, the few studies’ findings on the impact of competitive strategy on firm growth are controversial. These studies are mainly based on the resource-based theory, which explains that the availability of VRIN resources is an element that affects the growth of firms in the implementation of low-cost strategies and differentiation strategies [7, 8]. Instead, this paper considers the multifunctional characteristics of resources in firm growth theory [25]. We explore the relationship between competitive strategy and the growth of Chinese manufacturing firms from both resource and competitive perspectives. It also provides a new theoretical explanation for the relationship between competitive strategy and firm growth.

Second, based on institutional theory [15], this paper explores the moderating role of the development zone level and further uncovers the "black box" of how competitive strategy affects firm growth. It is found that firms in provincial development zones choose low-cost strategies that are more conducive to growth than national development zones. Firms in national development zones choose differentiation strategies that are more conducive to growth than provincial development zones. According to the firm growth theory and the competition theory, both low-cost strategy and differentiation strategy may provide a competitive advantage to the firm, which in turn promotes growth [4, 23]. However, given the different institutional environments in different regions in the Chinese context, there are differences in the level of resource support provided by national and provincial development zones. The relationship between competitive strategy and firm growth will change accordingly. On deeper analysis, firms in the national development zones have access to better quality resources to support them and increase innovation efforts under favorable institutional conditions. They build differentiation advantages that cannot be imitated by competitors by producing unique products or services. This makes the easier for them to survive and grow in competitive markets using differentiation strategies [10]. In contrast, firms in provincial development zones do not enjoy such intensity of policy incentives. They are devoting more energy to developing and integrating multifunctional resources, thereby capitalizing on cost advantages to achieve growth [31]. Previous articles have focused on the direct effects of development zones on firm growth, and few studies have compared national development zones with provincial development zones [17]. We investigate the moderating effects of different levels of development zones on the relationship between competitive strategy and firm growth from an institutional theory perspective. This enriches the study of institutional boundaries in the relationship between competitive strategy and firm growth.

### 5.2. Practical value

For firms, in terms of matching the competitive strategy with the level of development zones, the implementation of a low-cost strategy in provincial development zones is more conducive to growth than in national development zones. The implementation of a differentiation strategy is more conducive to growth in national development zones than in provincial development zones. National development zones and provincial development zones are established for different purposes and have different preferential policies [17]. National development zones can provide firms with high-quality resources and a favorable environment, while provincial development zones can provide firms with low-cost and non-market resources. Firms that have entered the development zone can adjust competitive strategies to ensure the maximum utilization of policy dividends. Firms can also choose the right development zone according to the situation of competitive strategy and choose a good external institutional environment to achieve growth more easily.

Our study also provides evidence for the government to formulate and optimize the policies of development zones. Differentiation and low-cost strategies have no advantages or disadvantages, and the successful implementation of either competitive strategy can bring competitive advantages to firms. The State Council increased guidance and incentives for innovation and technology exchange, thereby better facilitating the growth of firms implementing differentiated strategies in national development zones. Provincial governments provided a boost to reduce costs for firms implementing low-cost strategies within provincial development zones by providing suitable and favorable resources. In the long run, the implementation of a differentiation strategy is more beneficial to enhance the core competitiveness of firms and the country [34]. Local governments also need to optimize provincial development zone policies to assist in the growth of differentiated strategic firms. In short, the Government should create a favorable institutional environment through development zones. This could provide better resource support to firms implementing low-cost strategies and differentiation strategies to help them better achieve growth.

### 5.3. Limitations and future research

Our study still has limitations that deserve further exploration in the future. First, in terms of sample selection, this paper only selected Chinese manufacturing listed firms, which may affect the validity and generalizability of the findings. Future research can further expand the scope and time of the research subjects to explore the relationship of the variables more fully. Second, national development zones include economic and technological development zones, high-tech industrial development zones, and other types. Provincial development zones include economic development zones and industrial parks. Future research could examine whether there are differences in the moderating role played by these types of development zones in the relationship between competitive strategy and firm growth. Third, we have focused only on general firm growth. Firm growth also includes various types such as long-term growth and high-speed growth. The relationship between competitive strategy and the growth of different types of firms has not been well studied. Future research can further explore the mechanisms of competitive strategies on different types of growth to enrich the existing studies.

## Supporting information

S1 Data (XLSX)
